# Glucagon-like peptide-1 receptor agonists: a review from a cardiovascular perspective

**DOI:** 10.3389/fcvm.2025.1535134

**Published:** 2025-04-24

**Authors:** Yosra Turkistani

**Affiliations:** Department of Medicine, College of Medicine, Umm Al-Qura University, Makkah, Saudi Arabia

**Keywords:** cardiovascular disease, cardiovascular outcome, glucagon-like peptide-1 receptor agonists, heart failure, obesity

## Abstract

**Introduction:**

Glucagon-like peptide-1 receptor agonists (GLP-1 RA) are novel agents with proven cardiovascular (CV) benefits. GLP-1 RAs have been used for diabetes and found to improve CV outcomes in diabetic and nondiabetic patients. They are authorized for treating obesity. Our narrative review discussed the CV benefits of GLP-1 RAs in terms of controlling CV risk factors and improving CV outcomes in diabetic and nondiabetic patients regardless of their CV history, and the CV perspectives related to their use in clinical practice.

**Areas covered:**

Literature was searched with no limits on date or language, using various combinations of keywords. Data on the CV benefits of GLP-1 RAs and their use in clinical practice were summarized.

**Results:**

Several studies have discussed the CV beneficial effects of GLP-1 RAs in terms of reducing blood pressure, lipid levels, body weight, risk for arrhythmias, reducing the risk of major adverse CV events, and hospital admission for heart failure.

**Conclusion:**

The cardioprotective effects and low risk of hypoglycemia of GLP-1 RAs make them preferred agents in any multidisciplinary approach aiming to reduce CV disease burden and improve prognosis. Cardiologists are encouraged to strongly consider the CV benefits of GLP-1 RAs in their risk-reduction strategies.

## 1 Introduction

Besides the traditional standard-of-care agents, newer classes of glucose-lowering agents have been introduced and changed the landscape of type 2 diabetes mellitus (T2DM) management (1). Glucagon-like peptide-1 receptor agonists (GLP-1 RAs) are novel glucose-lowering agents that activate GLP-1 receptors leading to blood glucose reduction, postprandial glucose metabolism improvement, and gastric emptying delay (2). In response to ingested meals, GLP-1, an incretin hormone, stimulate glucose-dependent insulin secretion, suppress glucagon, prolong gastric emptying, and promote glucose uptake into muscles and adipose tissues (3). Consequently, the feeling of satiety is promoted and associated with a decrease of craving and food intake leading to weight loss. In addition, GLP-1 RAs decrease the glycated hemoglobin by 0.8%–1.5%, making them superior to other anti-diabetic drugs and a good choice for diabetes management (2). Currently, there are six FDA-approved GLP-1 RAs that have different properties, particularly in terms of administration, weight loss, and cardiovascular (CV) benefits: exenatide, liraglutide, dulaglutide, lixisenatide, semaglutide, and tirzepatide (4). Liraglutide, semaglutide, and tirzepatide are FDA approved for chronic weight management in patients with obesity or overweight with comorbidities (5).

The CV benefits were variable among the different available GLP-1 RAs; some randomized controlled trials demonstrated that liraglutide, dulaglutide, semaglutide, and albiglutide reduced the rate of CV events in T2DM patients (6–9), while other clinical trials concluded that lixisenatide and exenatide did not significantly improve CV outcomes in such patients (10, 11). In its most recent guidelines, the American Diabetes Association (ADA) established a comprehensive clinical approach to reduce CV risks in T2DM patients. This approach emphasized the use of cardioprotective GLP-1 RAs [liraglutide, dulaglutide, semaglutide, and albiglutide (withdrawn from the market for business reasons)] in T2DM patients with or at high risk for atherosclerotic CVD (ASCVD), heart failure (HF), or chronic kidney disease (12).

Another novel glucose-lowering agent is tirzepatide which has a dual mechanism of action: a GLP-1 and a glucose-dependent insulinotropic polypeptide (GIP) co-agonist. Both the GLP-1 and GIP receptors are expressed on neurons in the arcuate nucleus and other parts of the hypothalamus, and their activation reduce food intake and body weight (13, 14). A pre-specified meta-analysis including all the seven clinical trials that constituted the clinical development program of tirzepatide (SURPASS) demonstrated its CV safety in patients with T2DM in terms of not increasing the risk of major adverse CV events (MACE). This meta-analysis also suggested a non-significant trend toward potential CV benefits of tirzepatide (hazard ratios 0.8, [95% confidence interval (CI), 0.57–1.11] for MACE; 0.9 [95% CI, 0.50–1.61] for CV death; 0.8 [95% CI, 0.51–1.25] for all-cause death) in line with what was demonstrated for GLP-1 RAs (15).

These findings have promoted an interest to discover the CV benefits of this pharmacological class extending beyond its glycemic effect. GLP-1 RAs have been found to improve body weight, blood pressure (BP), lipid profile levels, and myocardial infarction (MI) outcomes even in nondiabetic patients without increasing the risk of hypoglycemic events (11, 16–20). Similarly, GLP-1 RAs have significantly reduced the risk of individual MACE components, all-cause mortality, and hospital admission for HF (21). The mechanisms underlying these CV benefits include promoting myocardial glucose uptake and utilization, reducing oxidative stress, inhibiting cardiomyocyte apoptosis, vasodilation, improving coronary blood flow, natriuresis, anti-inflammatory effects, renal protection, reducing plaque formation, and neurohormonal regulation (22). The anti-atherosclerosis and attenuated plaque formation effects of GLP-1 RAs are mediated through inhibiting the overall inflammatory response by suppressing the expression and release of proinflammatory cytokines (such as interleukin-6 and tumor necrosis factor-α) and suppressing the activation of nuclear factor-kappa B signaling pathway (23, 24). Another suggested mechanism for CV benefit is the reduction of epicardial adipose tissue which is involved in the development and progression of coronary artery disease, atrial fibrillation (AF), and HF. Epicardial adipose tissue responds to GLP-1 RAs in a mechanism that contributes to their CV benefits (25–28).

Our narrative review aimed to discuss the CV benefits of GLP-1 RAs in terms of controlling CV risk factors and improving HF outcomes, and the CV perspectives related to their use in clinical practice.

## 2 Methods

The authors conducted a literature search, with no limits on date or language, using various combinations of keywords, including “glucagon-like peptide-1”, “cardiovascular diseases”, “cardiovascular diseases prevention”, “cardiovascular outcome trial”, and “cardiovascular”. Further references were identified by searching the reference lists of retrieved articles and from the authors' knowledge of the field.

## 3 Results

### 3.1 Effects on overweight/obesity

Obesity has been identified as a chronic disease linked to increased mortality, coronary artery disease, T2DM, some types of cancer, and many other complications (29–31). A systematic review has showed that a 5%–10% weight reduction improves cardiometabolic parameters, including BP, blood glucose level and dyslipidemia (32).

A randomized controlled trial involving 3,731 patients who did not have T2DM showed that 3 mg of subcutaneous liraglutide was associated with body weight reduction. A total of 63.2% of the patients in the liraglutide arm lost at least 5% of their body weight compared with 27.1% in the placebo arm (*P* < 0.001). A weight loss difference of −5.6 kg (95% CI, −6.0 to −5.1; *P* < 0.001) was demonstrated at Week 56 in favor of liraglutide (33). These findings were further demonstrated in different meta-analyses (34–36). Liraglutide was well-tolerated, and the associated gastrointestinal adverse events were of mild to moderate severity. It is a short-acting GLP-1 RA and should be administered once daily.

Regarding exenatide, randomized trials reported that subcutaneous exenatide (10 mcg with a 4-week 5-mcg dose-initiation period) plus lifestyle modification resulted in weight reduction in nondiabetic obese patients (37). Meta-analyses of randomized controlled trials also showed that exenatide (10–20 mcg/day) could significantly reduce body weight in obese or overweight nondiabetic participants (34, 38). However, exenatide had the modest effect on weight reduction (39).

In the STEP-1 trial (phase 3, randomized, placebo-controlled), once-weekly injection of 2.4 mg semaglutide plus lifestyle modification were associated with sustained and clinically-relevant reduction in body weight in overweight or obese participants (40). A meta-analysis of 4 randomized controlled trials including a total of 3,613 nondiabetic individuals with obesity, reported a mean difference for weight reduction of −11.85% favoring semaglutide (95% CI, −12.81 to −10.90; *P* < 0.001). Most of the studies used once weekly subcutaneous injection of semaglutide starting at a dose of 0.25 mg and escalated every 4 weeks. Semaglutide was associated with gastrointestinal adverse events (nausea, vomiting, diarrhea, constipation) which were of mild to moderate severity, short duration, and resolved without treatment discontinuation (41).

Notably, a recent systematic review showed that compared to other semaglutide doses and GLP-1 RAs, 2.4 mg semaglutide had the greatest effect on weight reduction, controlling glucose level, and reducing BP; however, it was associated with the highest rate of gastrointestinal adverse events (39).

A systematic review and meta-analysis conducted to investigate the efficacy of tirzepatide on glycated hemoglobin, fasting serum glucose, and body weight in patients with uncontrolled T2DM reported that tirzepatide significantly improved glycemic control and body weight with an acceptable safety profile (42). In addition, SURMOUNT-1, a phase 3 clinical trial, evaluated the efficacy and safety of tirzepatide in nondiabetic adults with obesity or overweight. Three doses of once-weekly tirzepatide (5, 10, 15 mg) provided significant and sustained reduction in body weight (43).

The FDA has approved liraglutide (a maintenance dose of 3 mg injected daily), subcutaneous semaglutide (2.4 mg injected once weekly), and tirzepatide (5–15 mg injected once weekly) for chronic weight management in adults with body mass index (BMI) of 30 kg/m^2^ or greater, or with a BMI of 27 kg/m^2^ or greater with at least one weight-related condition (such as high BP, T2DM, or high cholesterol) to be used in addition to a reduced calorie diet and increased physical activity (44–46). However, exenatide was not approved for this indication.

Overall, robust findings have showed that GLP-1 RAs effectively promote weight loss. Weight reduction has been linked to improved CV risk factors and complications, particularly hypertension, ASCVD, HF, AF, pulmonary hypertension, and left ventricular hypertrophy (47, 48). In addition, weight reduction induced by GLP-1 RAs improved obstructive sleep apnea (another CV risk factor) severity and related outcomes (49, 50). Furthermore, GLP-RAs may be considered strong candidates for the management of nonalcoholic fatty liver disease looking at their promising results in reducing liver steatosis (51).

### 3.2 Effects on hypertension and hyperlipidemia

Besides their contribution to weight reduction and glycemic control, GLP-RAs were associated with BP and lipid levels reduction (52). The results of a recent meta-analysis showed that semaglutide, exenatide, and liraglutide were associated with a systolic BP (SBP) decrease ranging from—4.89 mm Hg to—2.68 mm Hg. Notably, only 2.4 mg subcutaneous semaglutide was associated with diastolic BP (DBP) decrease of 1.61 mm Hg (39). BP reduction has been shown to be associated with significant reduction in MACE (2). Moreover, hypertension is a common preventable risk factor for CVDs including coronary artery diseases, AF, and HF (53). However, a clinically meaningful reduction in BP which can be associated with lower risk of CV events was defined as a decrease of at least 5 mm Hg in SBP or 3 mm Hg in DBP (54).

Effects on lipid profile were collected as secondary outcomes from several clinical trials. GLP-1 RAs were associated with favorable effects on lipoprotein metabolism manifested as modest lowering of low-density lipoprotein, total cholesterol, and fasting triglyceride levels. Moreover, suppression of postprandial hypertriglyceridemia, a potential risk predictor of ASCVD, has been reported (20, 55, 56). Hence, GLP-1 RAs can alter the process of atherosclerosis and show atherothrombotic benefits; consequently, they decrease the risk of CVDs (2, 57, 58).

### 3.3 Effects on major adverse cardiovascular events (MACE)

GLP-1 RAs showed significant outcome reduction in terms of MACE reduction in both diabetic and nondiabetic patients regardless of their history of established CVD.

**Figure 1:**
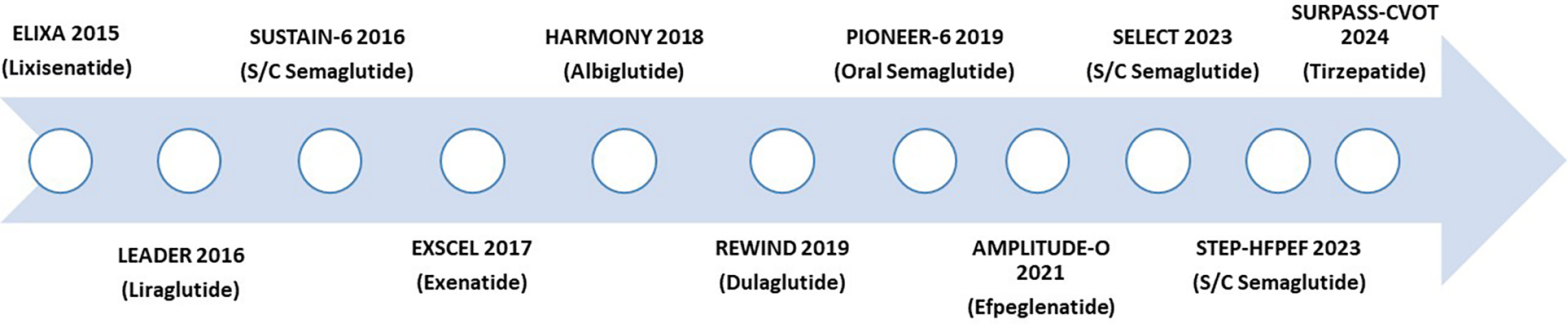
Timeline of GLP-1 RAs cardiovascular outcomes trials. GLP1 RA, glucagon-like peptide 1 receptor agonist; s/c, subcutaneous.

The CV outcome trials (CVOT) (Figure 1) of all the GLP-1 RAs (liraglutide, semaglutide, dulaglutide, albiglutide, extended-release exenatide, and lixisenatide) (6–11, 59) have demonstrated non-inferiority (CV safety). On the other hand, to date, three GLP-1 RAs have demonstrated a reduction in the MACE in patients with T2DM (liraglutide, subcutaneous semaglutide, dulaglutide) (60) (Table 1).

**Table 1:** Characteristics and results of GLP-1 RAs cardiovascular outcome trials.

Trial	Median follow-up time, year	Trial participants, *n*	Mean age, year	Female sex, *n* (%)	Established ASCVD, *n* (%)	Drug (dose) vs. placebo	CV composite outcome (CV death; nonfatal MI; nonfatal stroke)	*P*-value	Significant side effects
Elixa	2.1	6,068	60.3	2,894 (30.7)	6,068 (100)	Lixisenatide (10 µg SC daily)	13.4%	0.81	N/A
						Placebo	13.2%		
Exscel	3.2	14,752	62.0	5,603 (38.0)	10,782 (73.1)	Exenatide ER (2 mg SC weekly)	11.4%	0.06	Increased HR by 2.51 bpm
						Placebo	12.2%		
Harmony	1.6	9,463	64.1	2,894 (30.5)	9,463 (100)	Albiglutide (30–50 mg SC weekly)	7%	0.0006	None were significant
						Placebo	9%		
Amplitude	1.8	4,076	64.5	1,344 (33.0)	3,650 (89.6)	Efpeglenatide (4 or 6 mg SC weekly)	7%	0.007	Increased GI side effects
						Placebo	9.2%		
Rewind	5.5	9,901	66.0	4,589 (46.3)	3,114 (31.5)	Dulaglutide (1.5 mg SC weekly)	12%	0.026	Increased GI side effects
						Placebo	13.4%		
Leader	3.8	9,340	64.3	3,337 (35.7)	6,775 (72.5)	Liraglutide (1.8 mg SC weekly)	13%	0.01	Decreased incidence of renal or retinal microvascular events
						Placebo	14.9%		
Sustain-6	2.1	3,297	64.6	1,295 (39.3)	2,735 (83.0)	Semaglutide (0.5 and 1.0 mg SC weekly)	6.6%	0.02	Increased incidence of retinopathy
						Placebo	8.9%		
Pioneer-6	1.3	3,183	66.0	1,007 (31.6)	2,695 (84.7)	Semaglutide (14 mg oral daily)	3.8%	<0.001	Increased GI side effects
						Placebo	4.8%		
Select	3.3	17,604	61.6	4,872 (27.7)		Semaglutide (2.4 mg SC weekly)	6.5%	<0.001	Increased incidence of gallbladder disorders
						Placebo	8%		
Step HFPEF	1	529	68	297 (56.1)	98 (18.5)	Semaglutide (2.4 mg SC weekly)	+7.8 difference in KCCQ-CSS score, +20.3 m difference in 6-min walk distance change	<0.001	Increased GI side effects
						Placebo			

ASCVD, atherosclerotic cardiovascular disease; CV, cardiovascular; ER, extended release; GI, gastrointestinal; GLP-1 RA, glucagon-like peptide-1 receptor agonist; HR, heart rate; KCCQ-CSS, Kansas City cardiomyopathy questionnaire clinical summary score; MI, myocardial infarction; SC, subcutaneous.

The LEADER trial (6) investigated the CV effects of liraglutide in 9,340 T2DM patients who were randomized to receive either liraglutide (up to 1.8 mg once daily) or placebo. Patients included were either ≥ 50 or ≥60 years old with known CVD or multiple CV risk factors, respectively. The primary endpoint was three-point MACE composite outcome (first occurrence of death from CV causes, nonfatal MI, or nonfatal stroke) which occurred in 13% of the treatment arm vs. 14.9% of the placebo arm with a 13% relative risk reduction. Both non-inferiority (*P* < 0.001) and superiority (*P* = 0.01) to placebo were demonstrated for the primary composite MACE endpoint. The number needed to treat to prevent a MACE event was 53 over 3.8 years. The rates of nonfatal MI, nonfatal stroke, and hospitalization for HF were numerically but non-significantly lower in the liraglutide group vs. the placebo group.

Based on the demonstrated superiority, the FDA approved an additional labeled indication for liraglutide “to reduce the risk of major cardiovascular events in adults with type 2 diabetes mellitus and established cardiovascular disease” (61).

The CV outcomes of semaglutide in T2DM patients were evaluated in the SUSTAIN-6 trial (7). It included 3,297 T2DM patients who were on standard-of-care treatment and randomly assigned to receive once-weekly subcutaneous semaglutide (0.5 mg or 1.0 mg), or placebo for 104 weeks. Inclusion criteria were an age of ≥50 years with established CVD or an age of ≥60 years with at least one CV risk factor. The primary endpoint (three-point MACE composite outcome) was 6.6% in the semaglutide arm compared to 8.9% in the placebo arm (*P* < 0.001 for non-inferiority) resulting in a risk reduction of 26%. Semaglutide was significantly associated with a lower rate of nonfatal stroke (*P* = 0.04) and a numerically but non-significantly lower rate of nonfatal MI. Rate of CV death was similar in both arms. The number needed to treat to prevent a MACE event was 45 for 2 years. The superiority of subcutaneous semaglutide was demonstrated in a *post hoc* analysis for the primary composite MACE endpoint (*P* = 0.02). Hence, subcutaneous semaglutide labeling was updated to include an indication for reducing the risk of MACE in patients with T2DM and established CVD (61).

On the other hand, the PIONEER-6 trial aimed to evaluate the CV safety of once-daily oral semaglutide (the only oral GLP-1 RA) (59). It included 3,183 T2DM patients who were assigned to receive, in addition to standard of care, oral semaglutide (3–7 mg daily) or placebo with a median follow-up of 1.3 years. This trial showed that oral semaglutide was non-inferior to placebo and associated with a reduced risk of MACE composite outcome by 21% (*P* < 0.001 for non-inferiority) which was comparable to that reported by subcutaneous semaglutide in the SUSTAIN-6 trial. However, superiority with oral semaglutide was not achieved (*P* = 0.17). In both trails, sample size, discontinuation rate, mean diabetes duration, and percentage of patients with established ASCVD were comparable. The proportion of patients with chronic kidney disease was greater in the SUSTAIN-6 trial. Unlike subcutaneous semaglutide, more patients experienced nonfatal MI with oral semaglutide, but the oral formulation showed a statistically significant reduction in CV death and all-cause mortality. As a conclusion, oral semaglutide has a CV safety profile similar to that of the subcutaneous form, as shown in SUSTAIN-6 trial. Hence, it is unclear whether the lack of CV benefit with oral semaglutide is attributed to its formulation or secondary to selection bias. However, PIONEER-6 study was neither powered for superiority (CV efficacy) nor designed to determine statistical significance for secondary outcomes. In an attempt to assess CV efficacy of oral semaglutide after demonstrating its CV safety in the PIONEER-6 trial, a large CVOT (*N* = 9,650) is currently ongoing (SOUL trial) to compare the risk of MACE composite outcome with oral semaglutide vs. placebo in subjects with T2DM and established ASCVD and/or chronic kidney disease (62).

The CV outcomes of dulaglutide in T2DM patients were assessed in the REWIND trial (8). It included 9,901 patients with T2DM aged at least 50 years who had either an established CV event or CV risk factors. They were randomly assigned to receive either a weekly subcutaneous injection of dulaglutide (1.5 mg) or placebo. The primary endpoint was a three-point MACE composite outcome, and the tested hypothesis was superiority to placebo in terms of CV benefits. The primary composite outcome occurred in 12% of the dulaglutide group participants vs. 13.4% of the placebo group participants (*P* = 0·026). All-cause and CV mortality did not differ between groups. Dulaglutide was additionally approved for reducing the risk of MACE outcomes in T2DM adults with established CVD or multiple CV risk factors (61).

Several meta-analyses of the GLP-1 RAs CVOTs have been published (21, 63–65). A meta-analysis of eight GLP-1 RAs CVOTs demonstrated that GLP-1 RAs reduced the risk of MACE by 14% (*P* = 0.006) in the overall T2DM population. At the level of individual MACE components, GLP-1 RAs also reduced CV mortality (by 13%, *P* = 0.016), nonfatal stroke (by 16%, *P* = 0.007), and nonfatal MI (by 9%, but statistically non-significant).

On the other hand, another meta-analysis showed that GLP-1 RAs reduced the risk of CV death [Risk Ratio (RR), 0.90; 95% CI, 0.83–0.97; *P* = 0.004], and fatal or nonfatal stroke (RR, 0.85; 95% CI, 0.77–0.94; *P* = 0.001). However, GLP-1 RAs failed to reduce the risk of fatal or nonfatal MI (RR, 0.91; 95% CI, 0.82–1.01; *P* = 0.06) (66).

The reported CV benefits were not associated with an increase in risk of severe hypoglycemia, retinopathy, or pancreatic adverse effects (21).

Moreover, a very recent multicenter, double-blind, randomized, placebo-controlled trial (the SELECT trial), showed that weekly 2.4 mg subcutaneous semaglutide was superior to placebo in reducing the risk of CV death, nonfatal MI, or nonfatal stroke at a mean follow-up of 39.8 months. This trial included 17,604 nondiabetic overweight or obesity patients with preexisting CVD. The primary endpoint (a composite of death from CV causes, nonfatal MI, or nonfatal stroke) occurred in 6.5% of the semaglutide group vs. 8% of the placebo group (hazard ratio, 0.8; 95% CI, 0.72–0.90; *P* < 0.001) (67).

Based on the improved CV outcomes of GLP-1 RAs, the novel combination of GIP and GLP-1 RA may be another promising component in approaches aiming to reduce CV events, particularly in high-risk patients. A phase 3, randomized, double-blind CVOT, SURPASS-CVOT, is currently ongoing and aiming to assess tirzepatide vs. dulaglutide (1.5 mg weekly) for both non-inferiority and superiority in terms of CV outcomes (68). SURPASS-CVOT will provide definitive evidence on the CV safety and efficacy of tirzepatide.

### 3.4 Effects on heart failure

In addition to their demonstrated CV benefits, GLP-1 RAs have showed promising signals for their potential benefits in HF management. Two small clinical trials, the FIGHT and LIVE, evaluated the effect of liraglutide on patients with acute or chronic HF with reduced ejection fraction (HFrEF) (69, 70). Both trials failed to show any benefit of liraglutide in terms of HF outcomes.

On the other hand, the STEP-HFpEF trial evaluated the effect of semaglutide on nondiabetic obese patients with HF with preserved left ventricular EF (HFpEF) and obesity. Compared to placebo, semaglutide led to greater weight loss, HF-related symptoms improvement, reduced physical limitations, and significant difference in 6-minute walk distance (71). Of interest, the STEP-HFpEF trial has demonstrated benefits of semaglutide in nondiabetic patients with HFpEF and obesity. The effect of 2.4 mg once-weekly injected semaglutide in obese diabetic patients with HFpEF is currently investigated in an ongoing trial (STEP-HFpEF DM) (72).

No benefits in terms of HF outcomes were observed in the individual CVOTs of the GLP-1 RAs (22). However, Sattar et al. meta-analysis of the eight CVOTs including more than 60,000 patients with T2DM showed positive effect of GLP-1 RAs on hospital admission for HF (21). Similar results were also reported by the meta-analysis of Giugliano et al. (63). Overall, GLP-1 RAs showed a significant reduction in the risk of HF hospitalization by 10% (*P* = 0.023) to 11% (*P* = 0.013) (21, 63, 64).

On the other hand, a recent meta-analysis focusing on the effect of GLP-1 RAs on prognosis in patients with HF showed that GLP-1 RAs significantly reduced the risk of MACE compared to placebo in HF coexisting with T2DM. However, this benefit was not observed in all-cause death, hospitalization for HF, CV death, MI, stroke, and death or hospitalization for HF (65).

The benefits of GLP-1 RAs in HF may be explained in terms of their weight reduction properties. Obesity is linked to increased risk of HF incidence, particularly HFpEF (73). Moreover, the ability of GLP-1 RAs to reduce the generation of reactive oxygen species and systemic inflammation may be considered in this context (74).

### 3.5 Effects on arrythmia

GLP-1 RAs were associated with a significant reduction in all-cause mortality and CV mortality, and did not increase the risk of sudden cardiac death, atrial arrythmias, or ventricular arrhythmias in T2DM patients (75, 76). Although a meta-analysis including all the GLP-1 RAs CVOTs has showed that this group has no effect on the risk of various types of arrhythmias (77), two meta-analyses have demonstrated a reduced risk of atrial arrhythmias (78, 79). One of these meta-analyses compared the effect of different glucose-lowering agents on the risk of AF and included only studies that reported AF or atrial flutter as clinical end points with a follow-up period of at least 12 months (78). The second meta-analysis included randomized controlled trials (ELIXA, HARMONY OUTCOMES, PIONEER-6, SUSTAIN-6, LEADER) that compared GLP-1 RAs with placebo and met the critical criterion of a proportion of patients with T2DM and MI > 30% (79). Of interest, the subgroup analysis showed that semaglutide reduced the risk of atrial arrhythmias and AF by 36% and 38%, respectively, while no anti-arrhythmic effect was observed among the other GLP-1RAs (79). Hence, the anti-arrhythmic effects of semaglutide must be considered in the relevant clinical context i.e., in T2DM patient with MI. The favorable outcomes of semaglutide on AF was also emphasized by the findings of recent meta-analyses (80, 81), whether in patients with or without diabetes (81).

### 3.6 GLP-1 RAs in clinical practice

**Figure 2:**
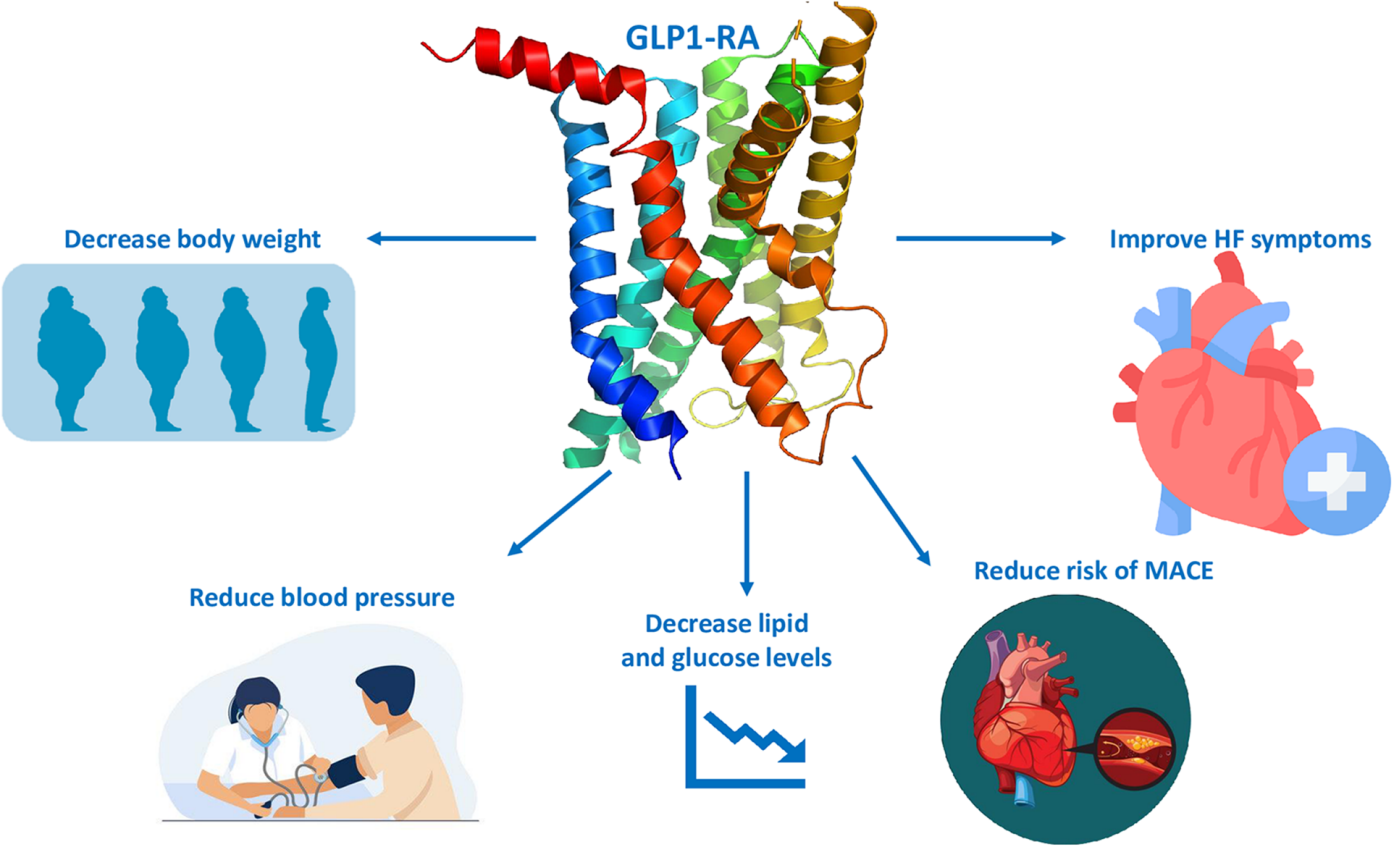
GLP-1 RAs cardiovascular benefits. GLP1 RA, glucagon-like peptide 1 receptor agonist; HF, heart failure; MACE, major adverse cardiovascular events.

The cardioprotective properties of GLP-1 RAs seem to be mediated through CV risk factors modification. GLP-1 RAs have been shown to improve glycemic control and insulin resistance; lower blood pressure; reduce body weight; and moderately reduce total cholesterol, low-density lipoprotein cholesterol, and triglycerides (82, 83) (Figure 2). However, the cardioprotective effects of GLP-1 RAs may be explained beyond traditional risk factors modification. GLP-1 RAs exert direct and indirect effects on the CV system including vasodilation, natriuresis anti-atherosclerotic effects, improvements in endothelial function, anti-inflammatory effects (decrease cytokines level such as tumor necrosis factor, interlukin-1, interlukin-6) and a consequent decrease in C-reactive protein, and reductions in infarct size (60, 84, 85).

Recently, GLP-1 RAs have been recommended by different guidelines. According to the 2023 ADA standards of care in diabetes (12), GLP-1 RAs with demonstrated CV benefit are considered for T2DM patients with established ASCVD or at high risk for ASCVD to reduce the risk of MACE. MACE reduction has been driven by the reduction of CV deaths as demonstrated with liraglutide, or reduction of nonfatal strokes as demonstrated with dulaglutide or semaglutide (6, 8, 62). Established ASCVD was defined as MI, stroke, any revascularization procedure, transient ischemic attack, unstable angina, amputation, symptomatic or asymptomatic coronary artery disease. Indicators of high CV risk included an age of >55 years, with two or more additional risk factors (obesity, hypertension, smoking, dyslipidemia, or albuminuria) (1). Similarly, the 2024 European Society of Cardiology (ESC) guidelines for the management of chronic coronary syndrome has recommended semaglutide for overweight (BMI >27 kg/m^2^) or obese chronic coronary syndrome patients without diabetes to reduce CV mortality, MI, or stroke. Moreover, GLP-1 RAs with proven CV benefits are recommended in patients with T2DM and chronic coronary syndrome to reduce CV event (86).

Cardiologists are invited to be more familiar with the members of this pharmacological family. All GLP-1 RAs including the dual agent tirzepatide and oral semaglutide demonstrated CV safety. Only liraglutide, subcutaneous semaglutide, and dulaglutide have showed CV efficacy. Furthermore, liraglutide, subcutaneous semaglutide, and tirzepatide are approved for chronic weight management regardless of diabetes status. These outcomes were further endorsed by the recent ESC Clinical Consensus Statement on Obesity and Cardiovascular Disease (87).

In addition, a recent analysis of the SELECT trial results showed that semaglutide use was associated with a substantial reduction in non-CV death. The lower rate of non-CV deaths was because of lower infectious deaths, in particular COVID-19–related deaths, suggesting that semaglutide has a favorable outcome in terms of mortality across a broad population of patients with CV disease and obesity (88).

Besides HbA1c and weight reduction, the criteria for initiating a GLP-1 RA with proven CV benefit should include an estimation of the patient's CV risk. Cardiologists may consider making an informed clinical decision by using reliable CV risk scores in addition to the judgment of available clinical evidence. The best-fit patients for GLP-1 RAs are those with ASCVD with a high risk of future stroke (e.g., with history of stroke or revascularization, or with evidence of significant artery narrowing). They may also be preferred in patients with elevated glycemia levels and high BMI aiming at modifying CV risk factors (2). Of note, GLP-1 RAs are not recommended for patients with T1DM.

GLP-1 RAs have a favorable safety profile. Gastrointestinal side effects are the most common including nausea, vomiting, and diarrhea. Unless combined with other agents that cause hypoglycemia, such risk is minimal with GLP-1 RAs. Pruritis and erythema at injection site may occur. Pancreatitis and pancreatic cancer were reported in some studies but not in large CVOTs with relatively long duration of follow-up. However, GLP-1 RAs are still not recommended in patients with a history of pancreatitis (2). Furthermore, GLP-1 RAs are not recommended for patients with severe gastrointestinal diseases (e.g., gastroparesis and inflammatory bowel disease); with a personal or family history of multiple endocrine neoplasia 2A, multiple endocrine neoplasia 2B, or medullary thyroid cancer (89). Subcutaneous semaglutide should be used cautiously in patients with pre-existing diabetic retinopathy (90, 91). It is noteworthy that GLP-1 RAs are contraindicated in pregnancy and breastfeeding; women who are planning to get pregnant should stop the medication 2 months before planned pregnancy (84). In addition, GLP-1 RAs were not associated with increased risk of suicide death, self-harm, or incident depression and anxiety-related disorders; suicide death among GLP-1 RAs users was rare (92).

Except oral semaglutide, GLP-1 RAs are administered subcutaneously, particularly those with proven CV benefits. Hence, treatment compliance can be improved by selecting agents that are injected once weekly such as semaglutide or dulaglutide. Gastrointestinal side effects are reduced by starting the GLP-1 RA at a low dose and titrating up over a certain duration according to each product labelling. Up-titration can be slowed to limit severity and frequency of side effects. Doses may be lowered temporarily until symptoms resolve. Upon initiation of a GLP-1 RAs and during up-titration, it is advisable for cardiologists to counsel their patients to take smaller meals, eat slowly and stop before feeling full, and avoid fatty or spicy foods (2).

## 4 Conclusion

GLP-1 RAs seem to emulate the evolution of statins and sodium-glucose co-transporter-2 inhibitors in terms of being approved for indications other than their initial indication and becoming promising agents in CV medicine which will continue to grow. They have emerged as a valuable therapeutic option for diabetic and nondiabetic individuals, particularly those at high risk of CVD.

GLP-1 RAs are the first weight-reduction medications that are safe and improve CV outcomes. The CV benefits of these agents are evident at the level of primary prevention of CVDs through controlling CV risk factors, particularly overweight and obesity. Moreover, the cardioprotective properties of GLP-1 RAs have been manifested in terms of reducing hypertension, hyperlipidemia, and cardiac arrythmias; controlling these factors plays a key role in the CV risk mitigating strategies. In addition to cardiometabolic risk factor reduction, their additional effect at the cellular and cytokines levels is likely to contribute to reduction of inflammation, atherosclerosis, and plaque formation.

The CV benefits extend to the promising effects in improving HF outcomes. Emerging evidence from clinical trials suggested a positive impact of GLP-1 RAs on nondiabetic obese HFpEF in terms of improving physical limitations, symptoms, and exercise function. These interesting findings may be also attributed to the weight reduction properties. Several CVOTs have demonstrated that GLP-1 RAs reduced CV risk in T2DM patients with established ASCVD or high CV risk independent of HbA1c levels.

Considering the reported CV findings, cardiologists are qualified to take the lead in adopting CV risk mitigations strategies as a part of comprehensive management strategies in collaboration with endocrinologists/diabetologists. GLP-1 RAs initiation by cardiologists in the appropriate clinical context may be considered a key factor in such strategies. Hence, implementing evidence-based agents with CV benefits by cardiologists is crucial for mitigating the CV risks in diabetic and nondiabetic patients. The cardioprotective effects of GLP-1 RAs along with their low risk of hypoglycemia make them preferred agents in any multidisciplinary approach for prevention of CVDs, reduction of the disease burden and improvement of prognosis. In this context, cardiologists are required to discuss with insurance companies the prescription of GLP-1 RAs and to advocate their use as cardioprotective gents in the presence of robust body of evidence. We are looking forward for the results of the SOUL and SURPASS-CVOT trials which, in case concluded with positive results, will add two new GLP-1RAs with CV efficacy.
